# Accessing Maternal Health Care in the Midst of the COVID-19 Pandemic: A Study in Two Districts of Assam, India

**DOI:** 10.3389/fgwh.2022.750520

**Published:** 2022-03-31

**Authors:** Rashmi Padhye, Anusha Purushotham, Maitrayee Paul, Nilangi Sardeshpande, Ramnath Ballala, Shelley Dhar, Sunil Kaul, Renu Khanna

**Affiliations:** ^1^Society for Health Alternatives (SAHAJ), Vadodara, India; ^2^Piramal Swasthya Management and Research Institute, Hyderabad, India; ^3^Institute of Development Action (IDeA) - The Ant, Guwahati, India

**Keywords:** COVID-19, maternal health, antenatal care, C-section, health services provision, expenditure on health services, stillbirth

## Abstract

**Background:**

COVID-19 pandemic and the subsequent national lockdown in India compelled the health system to focus on COVID-19 management. Information from the field indicated the impact of COVID- 19 on the provision of maternal health services. This research presents users' and providers' perspectives about the effect of the pandemic on maternal health services in select districts of Assam.

**Methods:**

The study was undertaken to understand the status of maternal health service provision and challenges faced by 110 pregnant and recently delivered women, 38 health care providers and 18 Village Health Sanitation and Nutrition Committee members during COVID-19 pandemic. Telephonic interviews were conducted with the users identified through simple random sampling. Healthcare providers and the community members were identified purposively.

**Results:**

Most of the interviewed women reported that they could access the health services, but had to spend out-of-pocket (for certain services) despite accessing the services from government health facilities. Healthcare providers highlighted the lack of transportation facilities and medicine unavailability as challenges in providing routine services. The study revealed high proportion of Caesarian section deliveries (42.6%, *n* = 32) and stillbirths (10.6%, *n* = 8).

**Discussion:**

This research hypothesizes the supply-side (health system) factors and demand-side (community-level) factors converged to affect the access to maternal health services. Health system preparedness by ensuring availability of all services at the last mile and strengthening existing community-reliant health services is recommended for uninterrupted good quality and affordable maternal health service provision.

## Introduction

The onslaught of the novel coronavirus disease 2019 (COVID-19) pandemic encumbered the health systems of countries across the globe. Some countries quickly adapted with 'extensive reorganization' of the health delivery system while others struggled (1). Variations in the intensity and duration were observed across the countries in the restrictions imposed for the movement of citizens (2).

Maternal health service provisioning was disrupted in several places as an effect of lockdown policies (1, 3). The negative effect of the COVID-19 pandemic on stillbirths, neonatal mortality, intrapartum care, and Cesarean section deliveries is predicted by various studies during the pandemic (4–6).

Restriction of movement due to the lockdown, absence of public transportation and fear of contracting COVID-19 infection kept women away from seeking service. These factors led to an estimated 20–50% decrease in access to critical maternal health care services in the Asia-Pacific region (7).

In India, the first case of COVID-19 was confirmed on January 27, 2020 and cases escalated during the next 2 months. The Union Government declared a national lockdown on March 24, 2020 to contain the infection. The historically under-resourced public health system in the country struggled to cope with the additional challenges posed by the COVID-19 pandemic, which impacted the provisioning of routine health services such as immunization or antenatal care. Most of the existing health infrastructure and human resources were engaged in managing the epidemic (8). Lacunae of the public health system, such as deficits in the infrastructure and disruption of maternal and child health services, particularly antenatal services and institutional deliveries got exacerbated (9–11) during this time. A study in tertiary level health facilities in Delhi, India, observed a 7.2% increase in high-risk pregnancies and 2.5 times increase in intensive care unit (ICU) admissions for pregnant women during the pandemic. This increase may be attributed to inadequate antenatal visits and delayed health-seeking due to the nationwide lockdown and fear of contracting the virus (12).

The Accredited Social Health Activist (ASHA) program has operated in the rural areas of India since 2005. ASHAs are trained female community health volunteers linking the community to the public health system (13). ASHAs have been instrumental in reaching out to the marginalized communities in their villages and enabling their access to maternal health services (14). Another village level structure for participatory planning and action on determinants of health (15, 16) at the village level is the Village Health Sanitation and Nutrition Committee (VHSNC), initiated in 2007 for monitoring the monthly Village Health Sanitation and Nutrition Day (VHSND) and the health services, particularly maternal and child health and nutrition services (17).

With the emergence of the COVID-19 pandemic, ASHAs were assigned to pandemic-related surveillance and contact tracing (9) activities that impacted their routine maternal health-related tasks (18). At the same time, VHSNC members' lack of formal training about their responsibilities and inadequate supportive supervision and monitoring hindered them from helping the community members during the pandemic in some places (19–21).

The present study has been conducted in Assam, which has historically performed poorly on maternal health indicators compared to other states in India. Assam has the highest Maternal Mortality Ratio (MMR) (215 per 100,000 live births in 2016–2018) (22) among the states, with only 64% of women reporting ANC registration during the first trimesters (23). One of the critical approaches to reducing maternal mortality is early identification of high-risk pregnancies (24). However, a study conducted in 2019–2020 revealed that in Assam only 7.36% of pregnancies were identified as high risk by the public health system (25).

Given these poor maternal health indicators of the state, this research was conceptualized to understand the impact of lockdown restrictions on the provisioning of maternal health services. This paper analyses the relationship between the supply- side (availability of services, skilled health care providers and infrastructure) and demand- side (barriers in accessing the health system, readiness of the patient to access the service given the higher risk of contracting the infection) (26) factors and their effect on the maternal and neonatal wellbeing during the COVID-19 pandemic in two districts of Assam.

## Materials and Methods

### Study Settings

The study was conducted in two districts of Assam, Kamrup (Rural) and Darrang. The districts were selected purposively based on the presence of grassroots civil society organizations working on maternal health issues. The two components of the study included 1. a telephonic questionnaire with a cross-sectional sample of pregnant and recently delivered women and 2. a purposive sample of health care providers and VHSNC members.

### Sampling Strategy and Data Collection

#### Pregnant and Recently Delivered Women

A simple random sampling was adopted to identify pregnant women meeting all of the following inclusion criteria: (a) Have registered phone numbers (belonging to themselves or their family members) in Government of India's Reproductive Child Health (RCH) portal in Darrang or Kamrup (Rural) districts (b) Have an estimated date of delivery between March 2020 and March 2021.

The database was accessible under the Early Childhood Development (ECD) call center operational under a public private partnership between the Government of Assam and one of the research partners.

From the study population of 6396 pregnant women, a sample size of 634 [452 from Kamrup (R.) and 182 from Darrang] was determined at 95% confidence level, 10% margin of error, anticipated frequency of 85% non-response rate (Supplementary Table 1). The non-response rate was estimated in accordance with the response rates of the routine ECD call centre programme data.

A structured telephonic questionnaire with close ended questions was pilot tested to assess the viability and efficacy of the process. The full questionnaire was administered to 171 respondents by a 6 member-research team to assess the administrative feasibility of the study including the resources necessary, technical capabilities of the research staff and data entry/data processing procedures. After pilot testing, the final telephonic questionnaire was administered by trained research assistants between October – November 2020. At the time of the interviews, three attempts were made to contact the participants over the phone before marking ‘no response.' The participant information sheet was shared orally and verbal informed consent was obtained from every respondent at the beginning of the telephone call, to confirm their participation in the study. Each interview on an average took 10 min. The data were captured on Microsoft Forms.

The telephonic questionnaire aimed to understand the status of maternal health service provisioning, including service utilization and out of pocket health expenditure. All the research assistants signed a non-disclosure agreement in accordance with the institutional data policy and only de-identified data were shared with the analysis team.

#### Health Care Providers and VHSNC Members

Staff members of the local organizations working on maternal health were trained on the interview guides. They conducted the interviews of a purposive sample of HCPs and VHSNC members from their field intervention areas. The selection was based on the availability and willingness to participate in the study, in the midst of the pandemic. All the respondents were interviewed upon seeking informed consent. Face -to-face interviews were conducted with 38 HCPs, including 15 health facility staff [13 Auxiliary Nurse Midwives (ANMs) and 2 Medical Officers (MOs)] and 23 ASHAs, and 18 VHSNC members from the two districts. They were interviewed during October and December 2020 in their villages with the help of interview guides designed to understand the effect of COVID-19 and the lockdown on the service provision (for the HCPs). VHSNC members were interviewed to understand their role, knowledge and capacity building efforts (if any) to provide access to maternal health services in their village. The average duration for the interviews was 15 min. The responses were noted by the interviewers and then translated and entered in Microsoft Excel.

#### Data Analysis

Microsoft Excel data outputs were used to generate data tables upon data cleaning and coding wherever required. Analysis was done using frequencies with percentages and cross tabulations (wherever possible). Further statistical analysis could not be done given- a. the small sample size and b. pre-coded answers due to the limitation of time while administering a telephonic questionnaire. For example, instead of actual expenses, they were recorded as a range. Qualitative description approach (27) was used to analyze the responses of the HCPs and the VHSNC members.

## Results

### Experiences of Pregnant and Recently Delivered Women

Out of the 634 women contacted telephonically, 150 women answered the call, and 114 women [64 from Kamrup (R.) and 50 from Darrang] consented to participate in the study. Fifty-two respondents were contacted on their phones, whereas 62 were contacted on phones belonging to their spouse, other family members, or neighbors.

The age of the respondents ranged between 18 and 39 years with a median age of 24 years. Thirty-one respondents were under the age of 20 years whereas 11 respondents belonged to the age group 31–39 years. 83% respondents (*n* = 95) belonged to the Below Poverty Level (BPL) category. All the respondents were registered as beneficiaries under Janani Suraksha Yojana (JSY), a central government scheme which provides conditional cash assistance for institutional delivery and post-delivery care.

#### Antenatal Care

One hundred ten respondents accessed ANC services. 83.6% (*n* = 92) received the recommended four or more ANC visits. 39.1% (*n* = 43) respondents received all 10 services whereas 55.5% (*n* = 61) received eight to nine services from the 10 listed services (Table 1).

**Table 1:** Access to ANC, delivery and PNC services for the pregnant and recently delivered women.

	**Number**	**Percent**
Respondents	114	100
**Antenatal care services**		
Women who received ANC^§^	110	96.4
**Number of ANC visits^†^**		
More than four times	60	54.5
Four times	32	29.1
less than four times	18	16.4
Number of ANC services^*^received by the pregnant women^†^		
All 10 services	43	39.1
8–9 services	61	55.5
5–7 services	3	2.7
<5 services	3	2.7
**Place of ANC**		
Government health facility*^†^*	104	94.6
ANC from Government health facility but ultrasound check-up and lab tests done from private health facility*^††^*	75	72.1
Private health facility	5	4.4
Home	1	1.0
Women who underwent ultrasonography as a part of ANC	81	73.6
**Delivery related services**		
Women who underwent deliveries^§^	75	65.8
**Pregnancy outcomes^§§^**		
Live births	63	84.0
Stillbirths	8	10.6
Neonatal deaths	4	5.3
**Place of delivery** ^§§^		
Government health facilities	58	77.3
Private health facilities	15	20.0
Home	2	2.7
**Post Natal Care services** ^§§^		
Women who received PNC	58	77.3
Women who received both ANC and PNC	54	72.0

* *The 10 ANC services include- 1) Urine Pregnancy Test (UPT) for confirmation of pregnancy; 2) testing for Hemoglobin (Hb) levels; 3) Blood Sugar levels; 4) checking the Blood Pressure (BP); 5) measuring Height; 6) measuring Weight; 7) Physical Examination; 8) Ultrasound check-up (USG) to check on the intrauterine growth of the fetus; 9) provision of Tetanus Toxoid (TT) Injection; and 10) Iron and Folic Acid (IFA) Tablets as per the recommended doses*.

† *The sample size used for the percent calculations is 110*.

†† *The sample size used for the percent calculations is 104*.

§ *The sample size used for the percent calculations is 114*.

§§ *The sample size used for the sample size calculations is 75*.

Although 104 women received ANC from a government health facility, 72.1% of them (*n* = 75) went to a private health facility/ laboratory for the ultrasound check-up and laboratory tests.

#### Delivery Care

65.8% (*n* = 75) of total respondents delivered during the reporting period. All except two had institutional deliveries. ASHAs conducted the two home deliveries. 77.3% (*n* = 58) were conducted in a government health facility.

The high proportions of stillbirths (eight stillbirths in 75 deliveries) and neonatal deaths (four neonatal deaths in 75 deliveries) (Table 1) were striking.

#### Post Natal Care

77.3% respondents (*n* = 58) received PNC. All except three women went to a government health facility or ASHA/ANM visited their homes for the postnatal check-up.

#### Expenses for Care

Services accessed from government health facilities are available for free or at a minimal monetary charge for the registration. The majority of respondents relied on government health facilities for the ANC services and no expenses were incurred by 27.3% respondents (*n* = 30). However, due to the unavailability of ultrasonography services in government health facilities, 72.1% respondents (*n* = 75) availed this service from a private health facility thus incurring out-of-pocket expenses. The details of category wise expenditure are given in Table 2.

**Table 2:** Expenses for ANC and delivery.

	**Number**	**Percent**
**Expenses incurred for ANC services** *^†^*		
More than Rs. 1,000/-	51	46.4
Rs. 501/–Rs. 1,000/-	26	23.6
Rs. 500- Rs. 100/-	3	2.7
No expenses	30	27.3
**Expenditure categories** *^††^*		
For ultrasound check-up	79	99
For laboratory tests	27	34
For medicines	12	15
For doctor's fees	8	10
For Transportation	8	10
**Expenses incurred for deliveries** ^§^		
More than Rs. 10,000/-	24	32
Rs. 5,001/–Rs. 10,000/-	19	25.3
Rs. 1,001- Rs. 5,000/-	13	17.3
Less than Rs. 1,000/-	3	4
No expenses	16	21.4
**Expenditure categories** ^§§^		
For medicines	50	85
For laboratory tests	36	61
For doctor's fees	24	41
For transportation	22	37
For blood transfusion	20	34

† *The sample size used for the percent calculations is 110*.

†† *The sample size used for the percent calculations is 80*.

§ *The sample size used for the percent calculations is 75*.

§§ *The sample size used for the percent calculations is 59*.

Sixteen respondents (two home deliveries and 14 deliveries in government health facilities) did not incur any expenses for the deliveries (Table 2). For the remaining 80.8% deliveries (*n* = 59), the expenses ranged from < ₹ 1,000 (~14 USD) to more than ₹ 10,000 (~40 USD), with 32% respondents (*n* = 24) reporting expenses above ₹ 10,000/- and another 25.3% (*n* = 19) reporting expenses between ₹ 5,000- and ₹ 10,000/- (~70 USD- 140 USD). Major delivery-related expenses were for medicines purchase (85%, *n* = 50) and laboratory tests (61%, *n* = 36). Although all the respondents were registered as beneficiaries for cash assistance under JSY (₹ 1,400 for institutional deliveries in rural areas and ₹ 3,000 for Caesarian section deliveries), the expenses incurred are much higher than the JSY benefits for most of the respondents.

#### Type of Health Facility, Type of Delivery and Delivery Expenses

The proportion of Caesarian section deliveries in both government (32.8%, *n* = 19) and private health facilities (86.7%, *n* = 13) is higher than the recommended Caesarian section rate considered by the WHO (10–15%) (28). Type of health facility and type of delivery both determined the delivery expenses (Table 3).

**Table 3:** Distribution of institutional deliveries according to the type of health facility, type of delivery and expenses incurred for deliveries.

	**Government health facilities^†^ (*n =* 58)**		**Private health facilities^§^(*n =* 15)**		Total^*^(*n =* 73)		
	**Vaginal deliveries number (%)**	**C-section deliveries number (%)**	**Vaginal deliveries number (%)**	**C-section deliveries number (%)**	**Vaginal deliveries number (%)**	**C-section deliveries number (%)**	**Total number (%)**
No expenses	10 (17.2)	4 (6.9)	0 (0.0)	0 (0.0)	10 (13.7)	4 (5.5)	14 (19.2)
Expenses below Rs. 10,000	26 (44.8)	9 (15.5)	0 (0.0)	0 (0.0)	26 (35.6)	9 (12.3)	35 (43.9)
Expenses above Rs. 10,000	3 (5.2)	6 (10.3)	2 (13.3)	13 (86.7)	5 (6.8)	19 (26.0)	24 (32.9)
Total	39 (67.2)	19 (32.8)	2 (13.3)	13 (86.7)	41 (56.2)	32 (43.8)	73 (100)

† *The sample size used for the percent calculations is 58*.

§ *The sample size used for the percent calculations is 15*.

* *The sample size used for the percent calculations is 73*.

In the government health facilities, 76% respondents (*n* = 44) reported to have incurred expenses ranging from < ₹ 1,000 (~14 USD) to more than ₹ 10,000 (~140 USD). All the deliveries in private health facilities (irrespective of the type of delivery) incurred expenses above ₹ 10,000 (~140 USD). For 9 out of 19 Caesarian section deliveries in Government health facilities, the respondents incurred expenses < ₹ 10,000 (~140 USD). Only four Caesarian section deliveries in the government health facilities incurred no expenses.

#### Effect of the Lockdown on Accessing Maternal Health Services

A very small number of women (7%, *n* = 8) reported challenges in accessing ANC or PNC services from health facilities due to the pandemic and the lockdown. Lack of transportation was the major challenge. Seven respondents stated that they could not avail services from their preferred government health facility due to the lockdown restrictions on travel, limited access to transport facilities, and unavailability of those particular health facilities for the delivery. Among them, three respondents delivered in a private clinic, one in a peripheral health facility, one at home and the remaining two had to travel to a higher-level health facility.

### Supply Side Issues—Perceptions of Health Care Providers

It was difficult for the Health Care Providers to continue providing the services because they feared contracting the virus. However, ASHAs and ANMs made home visits and coordinated with the pregnant women and their families over the telephone whenever required.

#### ANMs and Medical Officers–Challenges in Service Provision

According to the health facility staff (13 ANMs and two MOs), most ANC/PNC related service provisioning was managed through home visits. The staff faced issues in traveling to the villages for home visits because of a lack of travel options to reach remote areas. Even with the additional burden of COVID-19 related activities, the ANMs and doctors kept providing ANCs.

According to the interviewed providers, the non-availability of laboratory services was one of the significant gaps in the ANC provisioning. The village-level health centers were closed for 3 months during the lockdown. Post lockdown, the Iron and Folic Acid (IFA) tablets, an essential part of the ANC services, were unavailable at the village level health centers. These were available in select health centers but reaching these facilities was also difficult due to the lack of transportation.

Six ANMs reported managing high-risk pregnancies during the lockdown through home visits and regular follow-up over the phone. In one case, the woman delivered at home with support from ASHA, without any back up support of ANM or MO.

#### ASHAs' Role During the Pandemic and Challenges

ASHAs were asked about pregnancy related services and the challenges therein. Eighty-nine women were registered with these 23 ASHAs during this period. Eight ASHAs reported that they could not provide any of the expected health services during this period, while 13 ASHAs reported that all 60 pregnant women registered with them missed at least one ANC/PNC during this period. These numbers mean two-thirds (60 out of the total 89) of women registered under the 23 ASHAs missed at least one ANC/PNC during the lockdown.

ASHAs expressed the need for support from the health system and the ANMs and doctors for uninterrupted provisioning of the ANC/PNC in their areas during such unprecedented situations like the COVID-19 pandemic. They also talked about the making available the necessary set up for blood tests and contact details for an ambulance to carry the patients in emergency. Owing to the unavailability of IFA tablets, ASHAs emphasized the intake of iron-rich supplementary food items to the pregnant women. However, they also expressed concerns about the disruption of livelihoods and loss of income due to the lockdown and inability to get nutritious food for pregnant women from low income groups.

The ASHAs were not aware of the VHSNC members in their villages.

### Challenges in Community Participation—Roles of VHSNC Members

The VHSNC members reported that in the absence of village-level outreach services during the lockdown, they could not guide the pregnant women about the health care facility for their pregnancy related health care needs. VHSNC members were not aware about their roles and responsibilities in general and, more specifically, during the COVID-19 pandemic.

**Figure 1:**
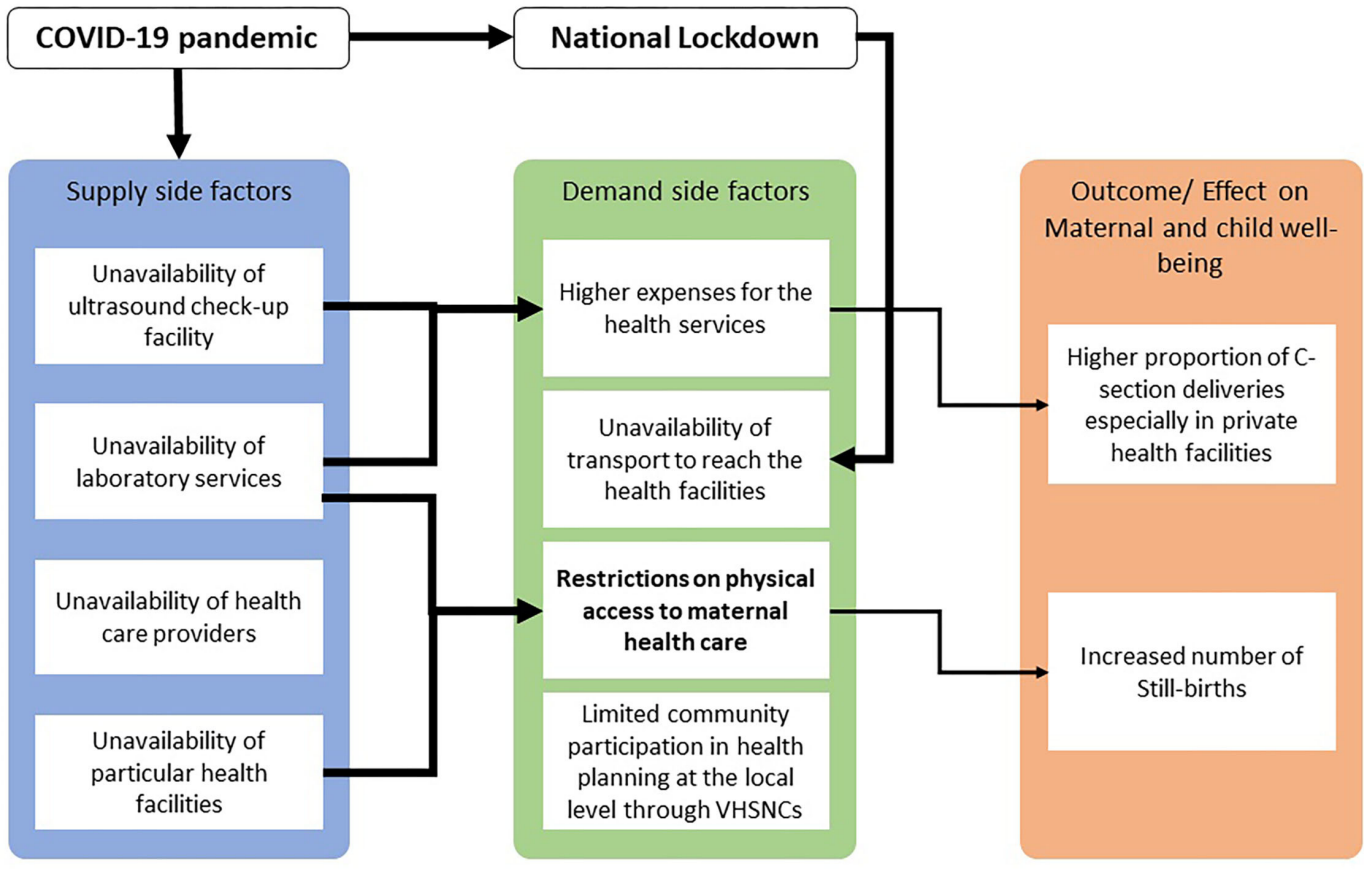
Empirical framework.

The research findings from both the demand and supply side can be summarized with the help of an empirical framework (Figure 1) that depicts the interrelations between supply-side and demand-side factors for access to maternal health services in these two districts of Assam.

## Discussion

COVID-19 pandemic and the subsequent lockdown changed very few things at the ground level for maternal health service delivery for these respondents. Most of the service delivery-related findings of this research are commensurate with Health Management Information System (HMIS) data from the previous year (2019-20). In the current research, 83.6% respondents (*n* = 92) received a minimum of four ANCs. HMIS data for Assam for 2019-20 (29) show similar findings, with 85.3% women receiving four or more ANCs.

One of the negative effects of the COVID-19 pandemic was unanticipated increased expenses for laboratory services and ultrasound check-up from private health facilities for respondents who had chosen a government health facility for ANC services. Unavailability of IFA tablets in village level health centres for Assam where 54.2% pregnant women are anemic (23) is also concerning.

Commensurate with both HMIS (2019-20) and NFHS-5 (2019-20) (23) findings from current research show high levels of institutional deliveries and deliveries done by the skilled birth attendants (SBAs). Despite this, it shows considerably higher proportion of stillbirths (8 from 75 deliveries) as compared to the Still Birth Rate for Assam in SRS Statistical Report 2017 (2 per 1,000 live births) (30). In addition four early neonatal deaths were also recorded from the 75 deliveries. A substantial indirect impact of COVID-19 on the perinatal outcome, including an increased rate of stillbirths, is also observed in different studies across different countries in the world, including India (6, 31, 32) but of a lesser magnitude. The rise in perinatal mortality could be linked with pandemic-related healthcare disruptions due to the movement restrictions during lockdown (3). Early identification of complications, availability of emergency obstetric services and prompt referral services help to avoid early neonatal deaths (33). Although an apparent link between the restricted physical access to the ANC and delivery services and the high levels of perinatal mortality (stillbirths and early neonatal deaths) could not be established from this research, it has underlined the need to study this further.

The reporting of Caesarian section deliveries (42.7%) is high as compared to recent state-level proportions from NFHS-5 (18.1%) and HMIS (23.5%). Also, majority of the deliveries in private health facilities (86.7%) were Caesarian section deliveries. It is well-established that the likelihood of Caesarian section delivery in a private health facility is higher than a public health facility regardless of other medical and economic factors (5, 34).

The difficulties in reaching the health facility due to lack of transportation during the pandemic are echoed in other studies for pregnant women in Panama and different states within India, viz., Chhattisgarh, Jharkhand, and Telangana (7).

ASHAs and VHSNC members are the official community representatives in the health system. The role of ASHAs in the improved utilization of ANC services, skilled birth attendance, and institutional births is highlighted in a recent study (14). Training of the local level health workers and community members would help manage primary treatment on the ground in such public health emergencies. For effective implementation of the health service delivery at the local level, these crucial stakeholders need to work in tandem.

The research has a limitation of small sample size from a localized area. Also, the most marginalized women with no access to phones could not be covered in this research. In addition, this study has not been able to show “true effect” of the pandemic on the maternal health outcomes. This observational study provides a framework for potential linkages between the maternal health outcomes and the COVID-19 pandemic and subsequent national lockdown for future research studies to explore. More research is also recommended to determine the causes of the rise in Caesarian section deliveries and to understand the causes behind stillbirths.

In conclusion, the COVID-19 pandemic has affected women's access to maternal health services in numerous indirect ways in select areas from two districts of Assam. The health system (supply-side) factors and the community level (demand-side) factors have worked together to affect the maternal and neonatal wellbeing. Strengthening the existing health system (26), providing sustained health service delivery at different levels for essential services, including maternal health services (35) and preparing the health system to deal with unprecedented situations (7) like the COVID-19 pandemic is recommended. Assuring transportation and a safe working environment for healthcare workers is recommended. For Assam, affordable and uninterrupted good quality maternal health service provision at all levels is recommended to reduce maternal mortality and improve other maternal health indicators.

## Data Availability Statement

The raw data supporting the conclusions of this article will be made available by the authors, without undue reservation.

## Ethics Statement

The studies involving human participants were reviewed and approved by Institutional Ethics Committee at SAHAJ and Piramal Swasthya Institutional Ethics Committee. Written informed consent for participation was not required for this study in accordance with the national legislation and the institutional requirements.

## Author Contributions

RP performed the data analysis and wrote the first draft of the manuscript. AP, SD, and RB prepared the study tools and coordinated the data collection of the quantitative section. MP coordinated the data collection activities on the field and supported data analysis from qualitative interviews. AP, NS, RK, and SK reviewed the first draft critically. All authors contributed to the conceptualization, study design, manuscript revision, read, and approved the submitted version.

## Conflict of Interest

The authors declare that the research was conducted in the absence of any commercial or financial relationships that could be construed as a potential conflict of interest.

## Publisher's Note

All claims expressed in this article are solely those of the authors and do not necessarily represent those of their affiliated organizations, or those of the publisher, the editors and the reviewers. Any product that may be evaluated in this article, or claim that may be made by its manufacturer, is not guaranteed or endorsed by the publisher.
