# Supplementary material for: Accessing Maternal Health Care in the Midst of the COVID-19 Pandemic: A Study in Two Districts of Assam, India
Source: Front Glob Womens Health. 2022 Mar 31;3:750520. doi: 10.3389/fgwh.2022.750520 (PMC9008699; doi:10.3389/fgwh.2022.750520)
Supplement: Supplementary file 1 [file Table_1.DOCX]

**Supplementary Table 1: Sample size calculation for the pregnant and recently delivered women**

| 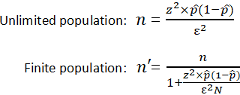  Z = Z-score for 95% confidence interval = 1.96  P = Population proportion = 0.5 (unknown proportion)  n = Sample Size for unlimited population  n’ = Sample Size for finite population  E = Margin of error = 10% or 0.10  N = Size of Finite Population = 8980    Steps of calculation  n = 1.96^2^ x 0.5 (1-0.5)/0.1^2^= 96.04  n’ = 96.04/(1+ ((1.96^2^ x 0.5(1-0.5))/(0.1^2^x 8980) = 95.089  Rounding down to the last decimal = 95  **Sample size = 95**  Non-Response rate = 80%  Adjusting for response rate = 95/1-0.8 = 475  **Adjusted Sample Size = 475**    Darrang Population: 3851  Kamrup (R.) Population: 5129    Darrang Sample Size: 204  Kamrup (R.) Sample Size: 271  **Total Sample Size: 475** |
| --- |
